# Rediscovering a Forgotten System of Symbiosis: Historical Perspective and Future Potential

**DOI:** 10.3390/genes11091063

**Published:** 2020-09-09

**Authors:** Vincent G. Martinson

**Affiliations:** Department of Biology, MSC03 2020, 1 University of New Mexico, Albuquerque, NM 87131-0001, USA; vmartinson@unm.edu; Tel.:+1-505-277-3711

**Keywords:** *Stegobium*, *Lasioderma*, Cigarette beetle, Drugstore beetle, *Symbiotaphrina*, fungi, Bostrichoidea, host–microbe

## Abstract

While the majority of symbiosis research is focused on bacteria, microbial eukaryotes play important roles in the microbiota and as pathogens, especially the incredibly diverse Fungi kingdom. The recent emergence of widespread pathogens in wildlife (bats, amphibians, snakes) and multidrug-resistant opportunists in human populations (*Candida auris*) has highlighted the importance of better understanding animal–fungus interactions. Regardless of their prominence there are few animal–fungus symbiosis models, but modern technological advances are allowing researchers to utilize novel organisms and systems. Here, I review a forgotten system of animal–fungus interactions: the beetle–fungus symbioses of Drugstore and Cigarette beetles with their symbiont *Symbiotaphrina*. As pioneering systems for the study of mutualistic symbioses, they were heavily researched between 1920 and 1970, but have received only sporadic attention in the past 40 years. Several features make them unique research organisms, including (1) the symbiont is both extracellular and intracellular during the life cycle of the host, and (2) both beetle and fungus can be cultured in isolation. Specifically, fungal symbionts intracellularly infect cells in the larval and adult beetle gut, while accessory glands in adult females harbor extracellular fungi. In this way, research on the microbiota, pathogenesis/infection, and mutualism can be performed. Furthermore, these beetles are economically important stored-product pests found worldwide. In addition to providing a historical perspective of the research undertaken and an overview of beetle biology and their symbiosis with *Symbiotaphrina*, I performed two analyses on publicly available genomic data. First, in a preliminary comparative genomic analysis of the fungal symbionts, I found striking differences in the pathways for the biosynthesis of two B vitamins important for the host beetle, thiamine and biotin. Second, I estimated the most recent common ancestor for Drugstore and Cigarette beetles at 8.8–13.5 Mya using sequence divergence (CO1 gene). Together, these analyses demonstrate that modern methods and data (genomics, transcriptomes, etc.) have great potential to transform these beetle–fungus systems into model systems again.

## 1. Modern Systems of Symbiosis Research

The current animal–microbe research landscape is dominated by the same model systems used in genetic and biomedical research (e.g., mice, zebrafish, *Drosophila melanogaster*, *Caenorhabditis elegans*) [1]. These systems provide resources and benefits not found in other species and have bolstered research on the microbiome, enabling key discoveries about mechanistic, ecological and evolutionary interactions with microorganisms [2,3]. While these traditional systems remain essential to this research, new systems are being developed to address specific questions and expand the diversity of experimental systems (e.g., *Apis*–microbiome, Cockroaches–microbiome, Heteropteran–*Burkholderia*, *Hydra*–bacteria systems) [4,5,6,7,8]. New organisms in animal–microbe research are emphasizing work on (1) pathogenic organisms, (2) mutualistic intracellular symbionts, and (3) the extracellular communities of microorganisms present on epithelia surfaces—known as the microbiome. While pathogens and mutualists reside on either end of the spectrum of symbiotic relationships, microbiome communities encompass diverse species that can have parasitic, mutualistic, commensal and neutral interactions with the host. Additionally, under different selective pressures or environmental conditions, any of these symbiotic interactions can change (e.g., with a high-sugar diet, a tooth-associated commensal may become opportunistically pathogenic; in the absence of a natural enemy, a protective mutualist may continue to take resources from the host and become parasitic), suggesting that the genes and gene products mediating interactions with the host share fundamental features. Indeed, comparative analyses in animal–microbe systems are identifying both similarities and differences in gene expression and immune system regulation among mutualistic and pathogenic relationships [9,10,11]. This not only provides an opportunity to find molecular pathways utilized in certain host–microbe relationships that have potential applications as targets in the control or elimination of pathogens, but also offers insight into the co-evolutionary processes that have shaped these relationships [12,13].

While bacteria remain the focus of most host–microbe research, microbial eukaryotes, especially fungi, are also common pathogens, endosymbionts, and members of the animal gut microbiota [14,15,16]. Novel and emerging fungal pathogens have led to drastic decreases and extinctions in wildlife populations of bats (White-nose syndrome [17]), amphibians (chytridiomycosis [18]), and snakes (*Ophidiomyces* [19]). This rising risk is not restricted to the animal kingdom—over two-thirds of agricultural crop diseases are caused by fungi and invasive pathogens threaten human food security [20]. Climate change has been implicated in this global pattern and directly linked to the rise of a multidrug-resistant opportunistic human pathogen, *Candida auris*, which appeared simultaneously on three continents between 2012 and 2015 [21]. Nonetheless, there are few systems for exploring host–fungi interactions.

Fungal diseases are estimated to cause 1.5 million deaths each year [22]. Additionally, over 1 billion people are affected by non-life-threating diseases including Athlete’s foot (*Trichophyton*), ringworm (*Microsporum*), histoplasmosis (*Histoplasma*), thrush (*Candida*), and vaginal yeast infections (*Candida*) that are caused by Ascomycota fungi [23,24]. Unlike bacterial pathogens, fungal diseases are often chronic and recalcitrant to therapy and overuse of drugs has led to a rise in resistance to common antifungals [25]. Many fungal species live as part of the human microbiota, inhabiting the gut or residing on skin, where they typically cause no disease and can protect against invasive pathogens through colonization resistance [15,26,27]. However, these close associates are also responsible for the majority of opportunistic infections, especially in people with decreased immune system function (elderly, HIV/AIDS, immunosuppressive therapies) [28]. Demographic changes and improvements to health care have led to an increase in susceptible populations and consequently a rise in the incidence of fungal infections worldwide over the past few decades, yet the fungal microbiota and fungal infections remain understudied relative to bacterial and viral threats [29,30]. Both maintaining an extracellular microbiota and preventing or controlling intracellular infection are critical to an organism’s survival; therefore, it is critical to identify how positive fungal associations are maintained and opportunistic fungal infections are controlled. Yet we lack an animal model that addresses these two host–microbe relationships simultaneously. Fortunately, a system of animal–fungal interactions utilized for over a century, but generally disregarded since the late 1980s has the unique ability to span research on pathogenesis, mutualism, and the microbiome. This system is the symbiosis between anobiid beetles and their fungal symbionts.

### Looking Back…to the Future

With modern genetic tools, researchers are increasingly able to forego classic model organisms and, instead, utilize new organisms/systems that are better suited to answer specific questions [1,31]. While this likely will lead to the emergence of novel model organisms, it is worthwhile examining the applicability of historical, overlooked research systems to take advantage of preliminary work that has already been performed. The technical limitations to research before advances in genetics and sequencing required model organisms to be easily maintained and manipulated. However, many of these models were left behind in favor of other systems, or after the end of a PI’s career [32].

**Figure 1:**
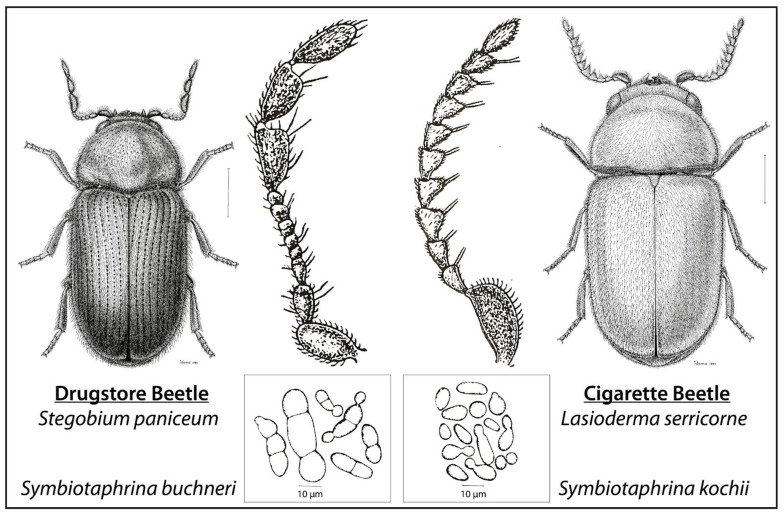
Drugstore and Cigarette beetles and their symbiotic fungi. Scale bar for beetles is 0.5 mm. Figure adapted from Bousquet (1990) [34], Jurzitza (1964) [35], Runner (1919) [36]. Reproduced with permission from Y. Bousquet, *Beetles Associated with Stored Products in Canada: An Identification Guide*; published by Canadian Governement Publishing Centre, 1990; illustrated by R. Idema.

**Figure 2:**
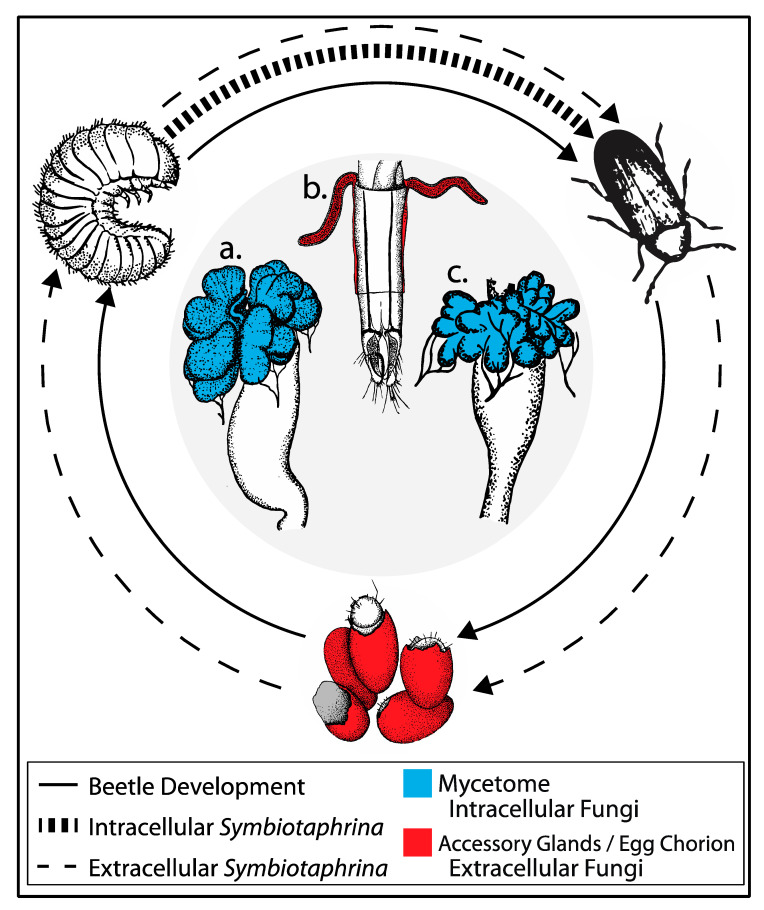
The life cycle of the beetle–fungus symbiosis between *St. paniceum* or *L. serricorne* and *Symbiotaphrina*. The fungal symbiont has extracellular and intracellular stages during the life cycle of their beetle hosts. Location and morphology of the larval midgut mycetome (**a**), adult female accessory gland (**b**), and adult midgut mycetome (**c**). The *Stegobium paniceum* mycetome with intracellular *Symbiotaphrina* in larva (**a**) and adult (**c**) is composed of deep evaginations (blue) located at the anterior midgut; posterior midgut (white). Accessory glands (red) attached to the oviposition device (white) hold extracellular *Symbiotaphrina* in the adult female beetle. Figure adapted from several sources: Egg—Buchner (1921) [37]; Larva—Buchner (1965) [38] & Koch (1933) [39]; Adult—White (1962) [40]; Mycetomes—Koch (1934) [41]; Accessory glands—Buchner (1965) [38] & Breitsprecher (1928) [42].

Along these lines, one of the oldest and most influential systems in microbial symbiosis research has been largely ignored for over 40 years: the beetle–fungus mutualism between Drugstore and Cigarette beetles with their symbiotic fungi, *Symbiotaphrina* (Figure 1). The fungal symbiont is both extracellular and intracellular during the beetle’s life cycle (covered in more depth below), located in gut-associated mycetomes in the larval and adult stages (Figure 2) [33]. Researchers have found that the fungus detoxifies compounds in the beetle diet and provides the host beetle with nutrients (vitamins, amino acids, sterols), although the exact metabolites exchanged differ between Drugstore and Cigarette beetles (see below). Although these two beetles are currently the only known hosts of *Symbiotaphrina*, this fungal symbiont may be more widespread, because very little research has been performed on species related to Drugstore and Cigarette beetles.

Instrumental in pioneering methods and theory around mutualistic host–symbiont associations, these fungal–beetle relationships are atypical among many host–symbiont systems in several aspects that include (1) the symbiont is eukaryotic not bacterial, (2) the symbiont has both intracellular and extracellular phases within the life cycle of the host, (3) the symbiont can be cultured on media separate from the host, and (4) the hosts can be maintained on enriched diet separate from the symbiont. Utilizing these unique features, their symbiotic relationships were explored continuously from 1920 to 1979.

This review will cover the evolutionary and ecological origins, the historical research performed, and the future potential as model organisms for fungi in the genus *Symbiotaphrina* and their hosts, the Drugstore beetle (*Stegobium paniceum*) and the Cigarette beetle (*Lasioderma serricorne*).

## 2. Drugstore and Cigarette Beetles—*Symbiotaphrina* Symbioses

### 2.1. The Beetles

**Figure 3:**
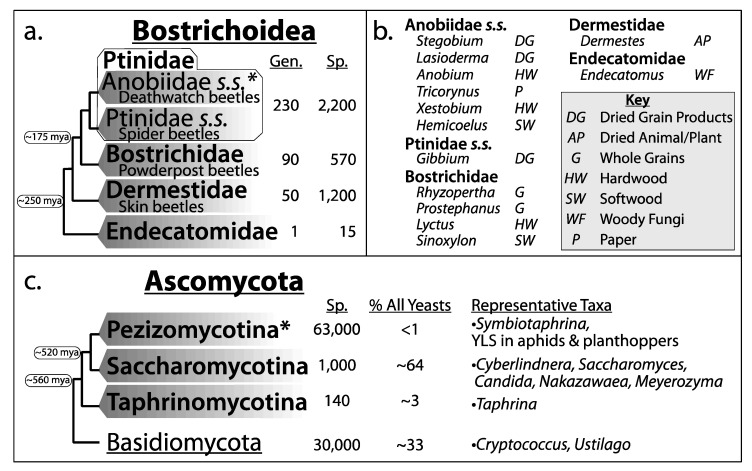
Phylogenetic relationships among beetles—Bostrichoidea (**a**); and fungi—Ascomycota (**c**). The estimated number of genera (Gen.) and species (Sp.) for each family of Bostrichoidea. For each subphylum of Ascomycota and the phylum Basidiomycota, the estimated number of species (Sp.), the fraction of all fungi with yeast-like growth (% all yeasts, e.g., 64% of all yeasts are found in Saccharomycotina), and representative taxa. Representative genera from each beetle family and their common diet (**b**). Asterisks indicate the taxonomic lineages containing the hosts (Drugstore and Cigarette beetles) and the symbiont (*Symbiotaphrina*). Figure adapted from several sources: beetle tree—Bell & Philips (2012) [46], Gearner (2019) [48], McKenna et al. (2019) [49]; fungal tree—Nagy et al. 2014 [50], Dujon & Louis (2017) [51], Kurtzman et al. 2011 [52], Shen et al. (2020) [53], Hibbett et al. (2018) [54], Spatafora et al. (2017) [55].

The Drugstore beetle, *Stegobium paniceum* (Linnaeus), and the Cigarette beetle, *Lasioderma serricorne* (Fabricius), are both members of Anobiidae *sensu stricto*, within the family Ptinidae *sensu lato* (*s.l.*—Latin meaning in the broad sense), which includes Ptinidae *sensu stricto* and Anobiidae *sensu stricto* (*s.s.*—Latin meaning in the strict sense). Ptinidae *s.l.* has approximately 230 genera and 2200 species divided between Ptinidae *s.s.* (spider beetles, subfamilies Ptininae and Gibbiinae) and Anobiidae *s.s.* (the remaining nine subfamilies of Ptinidae *s.l.*) distributed around the world (Figure 3a). Members of this family have received little study, possibly because of their conserved morphology, small size [43,44], commonly long generation times, and extremely varied habitats (e.g., dry wood, bark, seeds, pine cones, fungi, gall tissue on plants, animal dung) [45]. Likely many more species remain to be discovered, especially since there is evidence that localized endemic species may be common in this group [46]. Even with the lack of deep taxonomic work, these beetles have experienced frequent reclassifications. Initially recognized in the early 1800s, they were put into one family but have been repeatedly spilt and lumped under the names Ptinidae and Anobiidae. Most recently, these beetles have been united in the family Ptinidae [47].

Drugstore and Cigarette beetles (Figure 1) are both pests of stored products that have become associated with humans—for example, specimens of *L. serricorne* were unearthed inside the tomb of Tutankhamen, which places it in Egypt ~1500 B.C. [56,57]. *Stegobium paniceum* (syn. *Sitodrepa panicea*)—named in reference to its common collection in association with bread (Latin = pan)—is often referred to as the bread or biscuit beetle. Larval *St. paniceum* consume flour, pasta, dried spices, or other low-moisture content substrates including dried plants and herbs used for medicinal purposes, which is the origin of their common name—Drugstore beetle. *Lasioderma serricorne* (syn. *Ptinus serricornis*)—named in reference to its serrate antennae is a major pest of processed and unprocessed tobacco, hence its common name—Cigarette beetle.

Small, brown and with similar life histories (Table 1), these Drugstore and Cigarette beetles look similar without magnification. However, they have several distinct differences that suggest that they are not closely related. Differentiating these species can best be accomplished by examining the adults, where the antennae of *St. paniceum* are clubbed (final 3 segments have increased width, capitate) and *L. serricorne* are serrated (saw-like, serrate) (Figure 1). Additionally, the elytra (wing covers) of adult *St. paniceum* have rows of pits that give the appearance of lines (striated), whereas *L. serricorne* elytra are not striated (Figure 1). Outside of the context of mating and ovipositing, genitals are carried inside the adult body that otherwise lacks external sexual features, which impedes differentiating sexes in these beetles. In a common rearing environment and diet, females are larger in size and heavier than males, but reduced diet quality or larval crowding can obscure these differences [58]. *St. paniceum* males have a slot-like structure on the tarsal claws that females lack; however, this character can only be observed on slide-mounted specimens [59]. The only dependable way to discriminate males and females is at the pupal stage, when female genital papillae are divergent and protuberant (bulging outward), whereas male genital papillae are less pronounced and not protuberant [60,61,62].

Because of their agricultural and economic importance as destructive stored-product pests worldwide, Drugstore and Cigarette beetles have received much attention from entomologists about their evolutionary origins and their divergence [44,64,66]. Indeed, it has been hypothesized that these beetle species independently evolved to specialize on stored-product pests, *St. paniceum* from a wood-feeding ancestor, and *L. serricorne* from a plant product-feeding ancestor (possibly thistle) [33,67,68]. Researchers, investigating ways to control and monitor their populations in commercial settings, identified that they have different sex pheromones (stegobinone and serricornin), indicating a distant relationship [44]. Cytogenetic studies found both *St. paniceum* and *L. serricorne* to have 8 autosomes and 1 sex, for a 2n = 18 [69]. *L. serricorne* retains the ancestral achiasmate (XX-XY) coleopteran sex-chromosome system Xy_p_, but *St. paniceum* has lost its Y chromosome and instead has an XX-X0 sex-determination system. Genome sizes of *St. paniceum* and *L. serricorne* are estimated at 238–345 Mb, less than half the median (760 Mb) genome size among Coleoptera [70,71], similar to the Tsetse fly (366 Mb) [72], and much smaller than other insect models such as *Aedes aegypti* (1.3 Gb) [73]. Mitochondrial genomes are published for both beetle species [74,75], and full chromosomal genomes are in preparation [71]. Finally, while there have been few studies, phylogenetic evidence agrees that *St. paniceum* and *L. serricorne* are not sister species and might be separated by several million years [46,48].

#### Sequence-Based Estimate of Divergence Time for *L. serricorne*—*St. paniceum*:

Altogether the large differences between the beetle species in morphology (e.g., antennal shape, mycetome lobe number), chromosome-level sex-determination system, and phylogenetic unrelatedness [46,48], these species are likely quite genetically distant; however, this has not been estimated. In the absence of fossil-calibrated phylogenetic analysis, sequence-based estimates can provide a less conservative and rough range for the age of this beetle group and indirectly the symbiotic association with *Symbiotaphrina*. Using the publicly available mitochondrial genome sequences for these species, I estimated their divergence time using the “standard”, commonly cited insect mitochondrial clock rate of 0.0115/site/My (2.3% seq. div./My) and a more recent rate estimated in tenebrionoid beetles of 0.0177/site/My (3.54% seq. div./My) [76,77]. The full mitochondrial genomes of *St. paniceum* and *L. serricorne* share 75.5% identity, while the CO1 genes share 85.4% identity. The results from the CO1 gene (pairwise distance without evolutionary model correction) suggest a most recent common ancestor 8.8–13.5 Mya, confirming that the beetles are separated by several million years of evolution. However, a fossil discovered from mid-Cretaceous Kachin amber describing an Anobiidae *s.s.* species (possibly close to *Lasioderma*) dates to ~99 Mya, which may indicate a much older divergence between these beetles [78]. The anobiid-*Symbiotaphrina* symbiosis likely dates to before this common ancestor, since a non-symbiotic ancestor of *L. serricorne* and *St. paniceum* would require the independent acquisition of a *Symbiotaphrina* partner, which is an unlikely scenario. More work on both the beetle and the *Symbiotaphrina* phylogenies is required to firmly date this symbiosis and identify the number of independent symbiosis events between Anobiidae *s.s.* beetles and *Symbiotaphrina*.

### 2.2. The Fungi

Yeasts are a polyphyletic assemblage of fungi that spend all or most of their life cycle as single cells [50]. This form of growth is a convergent trait that has evolved independently in distantly related fungal clades and has hindered the taxonomic classification of yeast-like fungi including the symbiont of *St. paniceum* and *L. serricorne*, *Symbiotaphrina* (Figure 3c). Since the original discovery of *Symbiotaphrina*, taxonomists have found it difficult to categorize and consequently it has undergone several reclassifications [50,51,52] (Figure 3c). Initially, the *St. paniceum* symbiont was identified as a flagellate [79]; however, one year later it was successfully cultivated and revised as a yeast—provisionally placed within *Saccharomyces* [80]. Paul Buchner, often referred to as “the founder of systematic symbiosis research”, named the *St. paniceum* symbiont *Saccharomyces anobii* only to have it renamed in his honor as *Torulopsis buchnerii* [81]. The *L. serricorne* symbiont was initially cultivated by Pant and Fraenkel [82,83], who referred to the beetle fungi as “yeast-like symbionts” and noted that symbiont cells “differ from *S. cerevisiae* in that they cannot ferment glucose” and that “they have so far been classified only very imperfectly as belonging to the genus *Saccharomyces*” [83].

The 1960s–1970s saw a flurry of changes including the near-simultaneous, yet independent movement of both the *L. serricorne* and *St. paniceum* symbionts to the Taphrinales, and the introduction of the genus name *Symbiotaphrina* [84,85]. Standardized culturing methods found many metabolic differences between the two symbionts even with their close phylogenetic relationship (Table 2). The taxonomic names for the *St. paniceum* symbiont, *Symbiotaphrina buchneri* [84], and for the *L. serricorne* symbiont, *Symbiotaphrina kochii* [86], held until 1976 when the name *Torulopsis* was reestablished [87]. However, *Symbiotaphrina* was reinstated and validated four years later [88]. The arrival of molecular techniques revealed it was not a member of the Taphrinales, but instead related to filamentous ascomycetes [89,90]. Intron splicing patterns supported the placement of *Symbiotaphrina* in the subphylum Pezizomycotina, which accounts for the majority of Ascomycota fungi, but only as *incertae sedis* (Latin for “of uncertain placement”) [91].

The historical uncertainty surrounding *Symbiotaphrina*’s taxonomic placement has only recently been remedied with whole-genome sequencing. Now recognized as a member of Pezizomycotina class Xylonomycetes, *Symbiotaphrina* is most closely related to the endophytic fungi *Xylona heveae* and *Trinosporium guianense* (Figure 3c) [92]. Respectively, these fungi were isolated from rubber trees (*Hevea* spp.) and a wood-decaying polypore fungus (*Amauroderma* spp.) [93,94]. In addition, novel *Symbiotaphrina* species have been isolated or identified with sequence data from many plants (i.e., *Pinus*, *Picea*, *Populus*, *Acacia*, *Larrea*, *Adenocarpus*, *Quercus*, *Castanea*, *Descurainia*, *Dracaena*) across North America, Australia, Europe, and Asia [92,95]. These isolates tend to be found in decorticated xeric, sun-exposed decaying wood, but there is some evidence that they might survive endophytically in live plants, especially pines [95]. Signatures of a historical endophytic lifestyle similar to *Xylona heveae*, which has been hypothesized to be vectored between plants via an insect, are present in the *Sy. kochii* genome [92]. Specifically, both genomes harbor a similar repertoire of plant cell wall-degrading enzymes (e.g., pectinases, cutinases), which may help in evading plant defenses. Surviving within plant tissue has different pressures than surviving within beetle tissue and there may be clear patterns in gene degradation/loss, gene family expansion, or signs of selection.

The discovery of *Symbiotaphrina* in the environment, unconnected to beetles, suggests a large pool of free-living *Symbiotaphrina* (or close relatives) might be encountered by Ptinidae *s.l.* beetles in their natural habitat. Alternatively, these wood-associated isolates may be undiscovered beetle-associated symbionts left behind in frass or tunnel galleries. Additional surveys, genome sequencing, and phylogenetic analyses are required to determine the ecological interactions and transmission routes of wood- and beetle-associated *Symbiotaphrina*. However, culture-based evidence from the few isolates available reveals that while beetle symbionts are incapable of forming mycelia, conidia, and apothecia (sexual fruiting body), most wood-associated isolates can form these structures. These differences suggest that symbiont species have likely been exclusively associated with their beetle hosts for millions of years, which has potentially led to the loss of traits that may have inhibited survival within the host or transmission between generations (e.g., mycelia, conidia, and sexual reproduction) [95] (Table 3).

While genomes for both *Symbiotaphrina* species (~24 Mb each, Table 4) have been completed, they are highly fragmented because they were sequenced with short-read technology. Emerging long-read sequencing technologies (PacBio, Oxford Nanopore) will increase genome quality and adding genome sequences for closely related *Symbiotaphrina* species will provide insight into the evolution of this relationship and how genomes change in symbiotic association with a host.

#### Symbiotaphrina Diversity

Regardless of the large amount of work that has been performed on *St. paniceum* and *L. serricorne*, there has not been an analysis of symbiont diversity within an individual beetle, or across host populations. Intracellular symbionts that are inherited through the germ line generally have reduced diversity and are often individual strains, whereas microbiota communities that are extracellularly transmitted often have high diversity [96]. The *Symbiotaphrina* symbioses have similarities to both of these transmission modes making it unclear if they have high or low genetic diversity. Individual beetles may be infected at hatching with a *Symbiotaphrina* strain that remains associated with them through adulthood, alternatively cohabiting beetles may share symbionts and new infections may occur throughout an individual’s life, resulting in a changing community of symbionts similar to a microbiota. There is evidence that axenic larvae remain receptive to *Symbiotaphrina* infection throughout larval development prior to eclosion [83], making it possible that new symbionts might be acquired, and symbiont diversity may be high. Additionally, there is evidence of ecotypes within *St. paniceum* corresponding to diet (i.e., flour vs. tobacco) [60], which might hint that differences in the metabolic or detoxification potentials of symbiont strains mediate diet preference.

### 2.3. The Symbiosis

#### 2.3.1. Beetle–Fungus Life Cycle

The two beetles have largely similar life cycles and interactions with their fungal symbiont, with the one major difference being that *St. paniceum* spends nearly twice as long as passing through four larval instars than *L. serricorne*, with an average of 36 and 18 days, respectively (Table 1). Upon hatching, beetle larvae are symbiont-free and only after the oral uptake of *Symbiotaphrina* cells (located on the egg surface—or chorion) do the mycetocytes of the anterior midgut become intracellularly infected with the symbiont [37]. The mycetocytes are receptive to symbiont infection throughout larval development and are indistinguishable from epithelial gut cells for the first 5–7 days post hatch, after which they swell with fungal cells creating large evaginations in the anterior midgut (i.e., the mycetome) that remain intact through larval molts [83]. At pupation, while the majority of beetle tissues are reorganized during metamorphosis, the mycetome persists largely unchanged. It has been documented that fungal growth is increased within the pupal stage, when many fungi are found to have two buds per cell, whereas only single buds are observed in larval or adult *Lasioderma* mycetome fungi [83]. Upon eclosion, adult beetles harbor large intracellular symbiont populations which they have cultivated throughout larval and pupal development (Figure 2). In addition to the gut-associated mycetomes that harbor intracellular *Symbiotaphrina*, adult females have accessory glands, which are paired “pockets” near the ovipositor that contain large populations of extracellular *Symbiotaphrina* (Figure 2b). As an egg passes through the oviposition device, fungal cells are squeezed out of the accessory glands and deposited onto the chorion surface—these fungal cells are eventually taken up by the larvae to complete the life cycle. Little research has focused on accessory glands. In fact, it is not known how these organs are colonized by extracellular *Symbiotaphrina* [97]. Future work focused on how accessory glands are able to maintain large populations of extracellular fungi may provide clues for improving *Symbiotaphrina* cultivation conditions.

#### 2.3.2. Morphology of the Mycetome and Oviposition Organs

The structure of the anterior midgut evaginations differs in the two beetles, *St. paniceum* has four lobes, while *L. serricorne* has six lobes [83], which likely reflects genetic divergence (8–13.5 My). In both beetles, two of the six Malpighian tubules are attached anteriorly to the mycetome during larval instars, which might be involved with removal of symbiont-produced waste products that may be harmful if they pass through the beetle’s body cavity by hemolymph [33,98]. Alternatively, it has been suggested that the Malpighian tubules supply nitrogenous waste products to the symbiont to stimulate growth, as *Symbiotaphrina* isolates have been shown to utilize uric acid (commonly excreted by insects) [33,98]. Apart from these passing references there has been no work to explore these hypotheses, but future studies could investigate how host–symbiont co-evolution has mitigated toxic metabolites or other harmful byproducts produced by the fungal symbiont. The mycetome is also supplied with tracheae, suggesting that it may have an ample oxygen supply; however, no research has been performed to measure oxygen in the gut or the mycetome [98].

**Figure 4:**
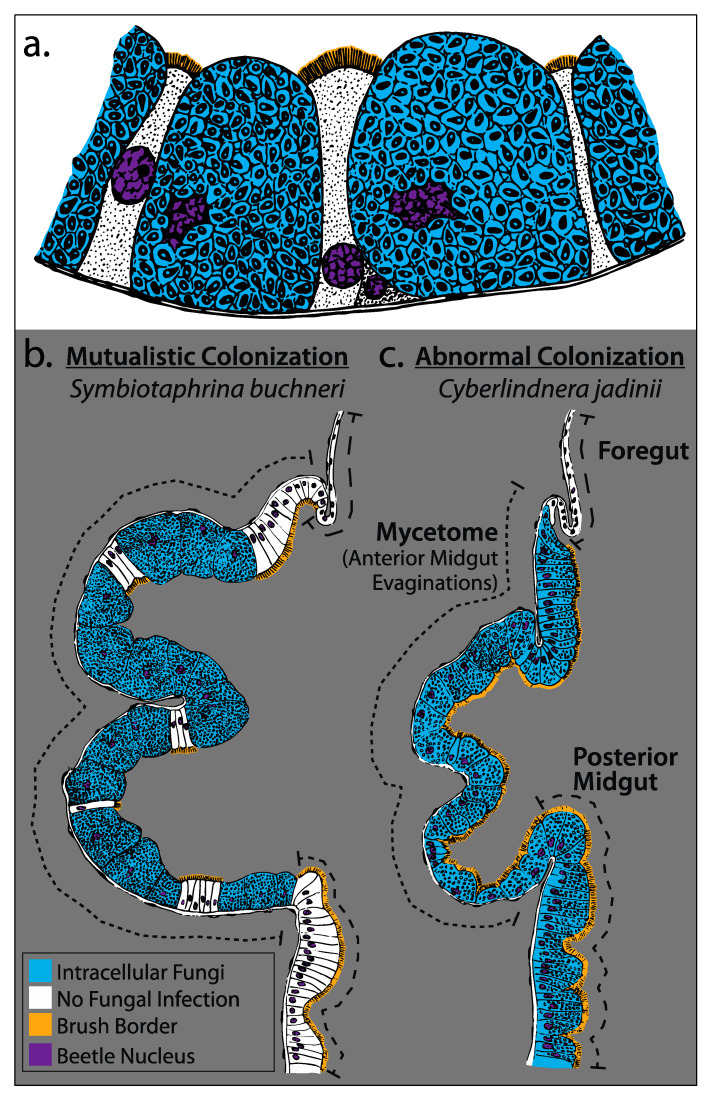
Cellular structure of the infection by fungi and in the midgut mycetome of *St. paniceum*. Within the mycetome, mycetocytes harbor intracellular *Symbiotaphrina* cells and lack a brush border, while pillar cells are not infected with fungi and retain a brush border (**a**). Intracellular infection of the mycetomes within the midgut evaginations of *St. paniceum* with its mutualistic partner—*Sy. buchneri* (**b**); and the free-living Saccharomyces—*Cy. jadinii* (**c**). In a mutualistic colonization, the intracellular fungi are restricted to the mycetome, whereas in the abnormal colonization by *Cy. Jadinii*, the intracellular fungi are found in midgut cells outside the mycetome. Figure adapted from Breitsprecher (1928) [42], Buchner (1965) [38], and Foeckler (1961) [103].

The midgut mycetome organ (Figure 2a,c and Figure 4a,b) is composed of two cell types: (1) mycetocytes—hypertrophied cells containing the fungal symbiont; and (2) pillar cells—uninfected cells that are small and slender with round nuclei similar to the midgut epithelial cells [37,38]. Upon symbiont infection, the brush border is lost in mycetocytes but is retained in the pillar and midgut cells. The ‘brush border’ refers to the microvilli covering the lumen-facing surface of gut cells, which is normally associated with enzyme production and nutrient absorption, but has also been shown to affect pathogen resistance [99,100]. The significance of eliminating the brush border is unknown but may be involved in recognizing the symbiont partner and preventing infection by non-symbionts, or with the physiological changes caused by intracellular fungal growth. Loss of a brush border in response to symbiont infection may be a conserved phenotype across ptinid species regardless of the identity of the symbiont. For example, in 1928 Breitsprecher observed that the brush border was lost in *Ernobius mollis*, which harbors the true yeast symbiont, first identified as *Candida*, but later reclassified using DNA sequence data as *Nakazawaea ernobii* [42,101,102]. However, very few studies have examined the fine-scale anatomy of anobiid–fungi interactions.

Electron microscopy surveys of mycetome ultrastructure in *L. serricorne* show that *Symbiotaphrina* are mostly located in basal regions of mycetocytes where the majority of fungal cells are viable, while cells appear to decrease in abundance and become lysed in the apical area (toward the gut lumen) [33,104]. These observations suggest that digestion of the symbiont may play a large role in nutrient acquisition (e.g., vitamins, sterols, amino acids) by the host beetle; however, there has been no follow up studies on the initial ultrastructural analysis [33].

The morphological changes that occur with symbiont uptake have received very limited study. In aposymbiotic beetles fed a diet enriched with yeast extract, the midgut mycetome develops normally (even after symbionts have been removed for several generations—passing comment by Koch); however, the mycetome is reduced in size relative to symbiont-harboring larvae and adults [105]. Further, there are no mentions in the literature of the accessory gland morphology in the absence of symbionts. More detailed physiological studies of the gut and mycetome may be able to identify differences induced by the symbiont (enzyme changes, pH changes, etc.) and how the mycetome develops (stem cells, regeneration).

#### 2.3.3. Gut Microbiota and Extracellular Symbionts

While the mycetome and accessory glands are critical for host–symbiont interactions in *St. paniceum* and *L. serricorne*, the gut also harbors *Symbiotaphrina* symbiont populations that have received little attention. Initially, symbionts are orally ingested by larvae, extracellularly recognized in the alimentary canal, and become intracellular within the midgut. Mycetome cells routinely lyse and pass through the larval gut, indicating that extracellular symbionts are common in the midgut and hindgut. While little is known about homeostasis and metabolism in the midgut of either *St. paniceum* or *L. serricorne*, they both utilize cryptonephridium to collect and recycle water from their low-water-content diets [33,98] and they both lack a peritrophic membrane as adults [106]. While adults consume little to no food, their guts have been found to contain intact midgut cells that have been expelled from the midgut wall, some containing the fungal symbiont [106]. This may suggest that digestion of the symbiont could contribute to adult nutrition, alternatively the expulsion of mycetocytes may be related to the colonization of the accessory glands by *Symbiotaphrina* and intergenerational symbiont transmission.

#### 2.3.4. Maintenance of an Infection through Symbiosis

Intracellular growth is normally associated with pathogens, where the innate immune system protects against invasion, or with long-term endosymbionts, where the loss of critical biochemical pathways prevents survival outside cells and symbiont growth is determined by host controls [107,108,109,110]. The more complex life history of the beetle–fungus relationship, which passes through both intracellular and extracellular phases, has likely been balanced through co-evolution of the host immune system and the symbiont metabolic factors [111]. There are few examples of extracellular/intracellular beneficial symbionts, and the beetle–fungus system provides a unique platform to address many questions: How are intracellular infections managed? What aspects of the host immune system are important? What prevents other fungi from entering the mycetocyte?

## 3. The Historical Research Perspective

The beetle–fungus symbiosis between *St. paniceum* and *Sy. buchneri* was first identified by W. Karawaiew in 1899 [79]. He meticulously illustrated the structure of the midgut mycetome, describing how uninfected cells were interspersed with cells infected with the symbiont but he erroneously placed the symbiont as a flagellate and suggested that it might be a parasite of the beetle. The following year, Karl Escherich, followed up on this work correctly classifying the symbiont as a yeast-like fungus, culturing the symbiont (although it is possible that this was a contaminating organism), and hypothesizing that the beetle–fungus relationship was not parasitic, but a mutualistic symbiosis [42,80,83,112].

First publishing on these beetles in 1921, Paul Buchner described the mode of transmission between generations for the *St. paniceum* symbiosis including the extracellular population of *Symbiotaphrina* in the accessory gland, depositing symbiont cells to the chorion of the egg, and the larval behavior of consuming the symbionts after hatching [37]. Along with Breitsprecher (1928) and later Gräbner (1954), Buchner showed that diverse bostrichoid beetles harbored fungal (and sometimes bacterial) symbionts in midgut mycetomes [37,42,81]. However, large groups of these beetles have yet to be surveyed for symbionts, particularly the Ptinidae *s.s.*

Anton Koch, a student of Buchner’s, advanced the experimental methods and performed many of the early studies on symbiosis in this system. Together, Koch and Buchner were able to manipulate the *St. paniceum*-*Symbiotaphrina* system in several foundational ways, developing methods to remove the symbiont from *St. paniceum* by surface-sterilizing eggs, and successfully rearing *St. paniceum* without its symbiont [97]. With these methods, Koch was able to learn the consequences of symbiont loss on host biology (delayed development or death), prove that reestablishment of the host–symbiont pair recovered the natural phenotype, and to pinpoint the metabolites capable of compensating for symbiont loss (B vitamins, sterols) [39,41,105].

Elaborating on the methods developed by Koch, Pant and Fraenkel performed a set of truly original experiments laying out the landscape of nutrient exchange between beetle and fungus across dietary conditions. After many failed attempts by other researchers Pant and Fraenkel successfully cultured both symbionts from the beetle species, which allowed them to perform symbiont exchange experiments [82,83]. One of the largest breakthroughs of this research was the formulation of a chemically-defined diet that had been adapted from previous experiments with *Tenebrio molitor* [113,114,115,116,117,118]. With this nutritional blueprint, both *St. paniceum* and *L. serricorne* were reared on a panel of diets excluding individual nutritional components or categories (e.g., vitamins, sterols) [82,83,119]. The contribution of symbionts was accessed by tracking aposymbiotic, homospecific, and heterospecific associations, which further accumulated evidence for the importance of symbiont-provisioned B vitamins as the basis of the host–symbiont mutualism. The following section will comment more on the heterospecific symbiont exchanges and the further expansion to non-symbiont fungi.

### 3.1. Nutritional Supplementation

Sterols: Sterols are required for animal growth and can be derived from animal sources (e.g., cholesterol), plants (e.g., ß-sitosterol, stigmasterol), or fungi (e.g., ergosterol) [120]. The development and survival rate of aposymbiotic beetles were moderately hindered by the removal of cholesterol from the diet [82,83,121]. While *St. paniceum* and *L. serricorne* may acquire plant sterols from their stored-product diets, they also likely obtain fungal sterols from their symbiont and the consequences of removing dietary sterol was much less than B vitamins.

Amino acids: Challenging the view that B vitamins were central to the symbiosis, experiments looking at the effects of protein-removal were performed with chemically defined diets deficient in single amino acids [119,122]. The results from rearing control beetles and aposymbiotic beetles on different diets clearly demonstrated that the symbiont provisioned essential and non-essential amino acids. This was among the first studies to show that intracellular symbionts could contribute to host amino acid requirements [119]. Culture-based assay of symbionts found that they were capable of utilizing inorganic sulphate in the biosynthesis of methionine and cysteine, yet another benefit provided to the host beetles in their often low protein diets [122,123].

B vitamins: The importance of B-group vitamins provided by the symbiont was evident from early studies on both *St. paniceum* and *L. serricorne* in dietary elimination studies [39,41,82,105]. In reciprocal symbiont transplant experiments (covered below), regardless of host species, *Sy. kochii* was able to sustain beetle growth in diets lacking thiamine and biotin, while *Sy. buchneri* was not [83]. This discovery was the first indication that the symbiont species differed in metabolic potential. When both were assayed in pure culture, the results agreed with the transplant experiments, finding that *Sy. kochii* was able to grow in the absence of biotin and thiamine, but *Sy. buchneri* was not [84]. Together, these studies provide strong evidence suggesting that *Sy. buchneri* lacks thiamine and biotin production. I hypothesize that genes in *Sy. buchneri*’s thiamine and biotin biosynthesis pathways are missing or have become pseudogenes, similar to many bacterial intracellular symbionts [124,125,126].

Comparative genomic analysis of *Symbiotaphrina* B-complex vitamin biosynthesis pathways:

In an attempt to use genomics to understand the underlying mechanisms differentiating the two *Symbiotaphrina* symbionts, I performed a preliminary comparison of the symbiont genomes focused on the biosynthesis of the B-complex vitamins, thiamine and biotin. These were selected because bioassays on isolated symbionts determined striking differences between *Sy. kochii* (thiamine and biotin prototroph) and *Sy. buchneri* (thiamine and biotin auxotroph), which suggests underlying genetic differences [84].

Within Xylonomycetes, genomes are available for *X. heveae*, *T. guianense*, and both *Symbiotaphrina* symbionts. The *T. guianense* and *Sy. kochii* genomes were used in the comparative genomics presented in the *X. heveae* genome paper, but the *Sy. buchneri* genome had not been analyzed [92]. Proteins for each genome was functionally annotated with the HMM-based KEGG ortholog assignment tool KofamKOALA [127] and mapped to metabolic pathways with KEGG Mapper [128]. Xylonomycetes genomes are generally similar in size, GC content, and total predicted gene number, even though they were sequenced by different agencies and annotated with different programs (Table 4). While the pathways for thiamine and biotin synthesis are not fully understood in Ascomycota and differ across fungal diversity [129], this preliminary comparative analysis identified differences between *Sy. buchneri* and *Sy. kochii* that likely explain their dissimilarity in vitamin production.

**Figure 5:**
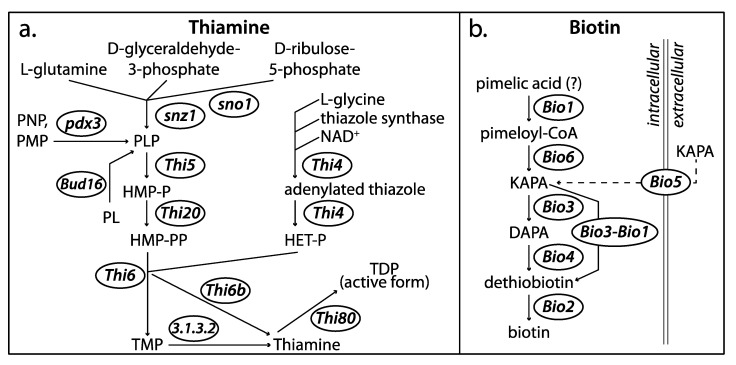
Biosynthesis pathway for the B vitamins thiamine (**a**) and biotin (**b**) in *Saccharomyces cerevisiae*. Figure based on Perli et al. (2020) [129].

Orthologs of the *Saccharomyces cerevisiae* thiamine biosynthesis pathway genes were identified in all Xylonomycetes genomes (Table 5, Figure 5a), with one exception: the Thi5 gene is absent from *Sy. buchneri*. Knockout of Thi5 in *S. cerevisiae* causes thiamine auxotrophy and indicates that Thi5 absence is likely the genetic cause underpinning *Sy. buchneri*’s lack of thiamine production [130]. Whereas *S. cerevisiae* harbors genes for the de novo biosynthesis of biotin (Bio1, Bio6, Bio3, Bio4, Bio2), Xylonomycetes likely import the intermediate KAPA with a putative membrane transporter Bio5, bypassing Bio1 and Bio6. Further simplifying the pathway, the fused gene Bio3-Bio1 performs the functions of both Bio3 and Bio4. While *Sy. kochii* has retained the biotin synthesis genes found in other Xylonomycetes, *Sy. buchneri* lacks both the Bio3-Bio1 and Bio2 genes, indicating that it is unable to produce biotin (Table 5, Figure 5b). Accordingly, genomic analyses confirm that *Sy. buchneri* has missing vitamin biosynthesis genes relative to *Sy. kochii* and their differences in vitamin production are not based on differential gene expression. However, the evolutionary forces that resulted in the loss of these genes have yet to be addressed.

*St. paniceum*’s hypothesized dietary shift from dry wood to stored grain products, which are high in vitamins, may have produced an environment conducive to *Sy. buchneri*‘s loss of the costly biosynthesis pathways for thiamine and biotin by way of eliminating the genes Thi5, Bio3-Bio1 and Bio2. For example, while Thi5 is required for thiamine biosynthesis it is also a metabolically costly suicide enzyme that must be replaced anew each reaction [131,132]. In *S. cerevisiae* strains, Thi5 has the most variable copy number among vitamin synthesis pathway genes [129], which might be due to the frequent loss of Thi5 in environments where thiamine can be readily scavenged and de novo vitamin synthesis is a competitive disadvantage.

Genome degradation (e.g., pseudogene formation, gene loss) that results in reduced genome size is commonly observed in bacterial symbionts as a result of genetic drift and Muller’s ratchet (elevated fixation of deleterious mutations in asexual populations) [107]. A host-associated lifestyle (especially intracellular growth) can reduce the effective population size (N_e_) of symbionts which increases genetic drift and relaxed selection on many symbiont traits (now provided by the host) can accelerate the effects of Muller’s ratchet [133]. This pattern has not been described in fungal symbiont genomes, and those that have been sequenced are similar in size to free-living relatives, including *Symbiotaphrina* (Table 4) [134,135,136]. Unlike bacterial symbionts, fungal symbionts may have increased recombination or sporadic sexual reproduction that reduces the effects of Muller’s ratchet and prevents genome size reduction. Alternatively, it has been hypothesized that eukaryotic genomes may increase in size when experiencing small N_e_ because of the expansion of mobile genetic elements (e.g., transposons, introns); however, this proliferation of mobile DNA may still create pseudogenes by interrupting genes [137]. Future genome sequencing and comparative analyses of free-living *Symbiotaphrina* isolates (Table 3) may shed light on the forces underlying genome sequence evolution in eukaryotic symbionts.

### 3.2. Detoxification

Detoxification of dietary components and drugs by the microbiome has received considerable attention in the past decade because of the importance that gut communities play in human-prescription drug interactions [138]. Additionally, the discovery that gut-associated microbes mediate the detoxification of creosote for desert woodrats has highlighted the manifold changes to ecology and behavior that microorganisms can have on their host [139]. In the insect world, substantial notice was granted to the discovery that insecticide resistance is linked to the metabolism of gut-associated symbionts in the bean bug *Riptortus pedestris* [140]. However, long before any of these discoveries, researchers studying *Symbiotaphrina* in the late 1980s were the first to find microbiota-assisted detoxification of many compounds, including common components of their host beetles diet [141].

It was hypothesized that *Symbiotaphrina* aided in its host beetle’s ability to survive on nicotine-rich tobacco, a very toxic compound for most insects, [98,142]. However, *Sy. kochi* growth is inhibited by 2–4% nicotine media (below levels reached in some tobacco cultivars) [142] and aposymbiotic beetles perform similar to symbiont-associated beetles on nicotine containing diets [33]. However, nicotine is only one compound in their diverse dietary range. Utilizing cultured isolates of *Sy. kochii*, Dowd and Shen at the USDA tested their abilities to degrade numerous chemicals and showed that *Sy. kochii* can not only detoxify plant secondary metabolites possibly encountered in the host diet (flavonoids, phenolics, cyanogenic glycosides), but also detoxifies insecticides (diazinon, malathion) and herbicides (glyphosate, 2,4-D) [143,144]. The fungus was able to utilize many toxin categories (mycotoxins, insecticides, herbicides, plant allelochemicals) as carbon sources by producing several detoxification enzymes (ester hydrolases, glucosidases, phosphatases, glutathione transferases) [145]. Additionally, aposymbiotic beetles treated with these toxins did not survive as long as symbiont-associated beetles, indicating that the activity is biologically relevant for host beetle survival [146]. Genomic insight may further enlighten the broad-spectrum detoxifying capabilities of *Symbiotaphrina* and related Xylonomycetes by identifying the genes/operons responsible and the evolutionary processes that have preserved these genes within this system. These genes may be targets for biotechnological use in the environmental remediation of areas containing diverse toxins.

### 3.3. Artificial Host–Symbiont Pairs

Nearly unique among obligate, intracellular symbiosis research systems, the ability to independently rear host beetles and cultivate fungal symbionts allows the experimental manipulation and creation of novel host–symbiont pairs. Early researchers first used this to produce reciprocal transfers among known symbionts, but later to branch out to create completely artificial symbioses. Francis Foeckler, a student of Buchner’s, became quite creative with joining hosts and symbionts, creating novel pairs where the fungal partner was a symbiont of distantly related cerambycid beetles or was a completely free-living yeast. The outcomes of these experimental pairs can be summarized into three groups based on the resulting mycetome structure: normal colonization, failure to colonize, and abnormal colonization.

Normal Colonization by Closely Related Symbionts: Reciprocal exchange of symbionts between *St. paniceum* and *L. serricorne* resulted in successful infection in the heterologous pairings and led to the differentiation of the mycetocytes and the loss of the brush border [83]. Yet, these symbionts did not perform equally in the host beetles, because of their differences in thiamine and biotin production (described above) [84]. The effect of symbiont species is only evident with nutritionally sparse diets; beetle development proceeds without issue on a nutritionally complete laboratory diet. These studies were only maintained for a single generation, and many questions remain unanswered. For example, it is unclear if the homologous symbiont can displace the heterologous symbiont.

Failure to Colonize by Distantly Related True Yeasts: Foeckler’s experiments attempting to create artificial host–symbiont associations by introducing fungi distantly related to *Symbiotaphrina* largely failed. Both free-living and insect-symbiotic fungi were tested for their ability to form associations with *St. paniceum* [103]. The symbionts used for this study were isolated from cerambycid beetles, which have independently forged symbiotic associations with yeasts and also harbor midgut-located mycetomes to house symbiont cells [147]. Specifically, an unknown fungal symbiont of *Spondylis buprestoides*, and *Candida rhagii* strains from *Rhagium bifasciatum* and *Rhagium inquisitor* were assayed for colonization of *St. paniceum* mycetomes. The free-living fungi explored were restricted to those that grow asexually with only single-celled, yeast-like cultures. Focus on yeast-like fungi—mainly true yeasts in the Saccharomycotina—followed the logic that all symbiotic fungi associated with beetles have yeast-like growth [147]. Among fungi, yeast-like growth has evolved independently several times from the ancestral mycelial/spore-forming fungi (Figure 3c) [50]; all known symbiotic fungi of insects share this trait. Yeast-like growth is suggested as “ideal for animal symbiosis” [147], since it does not require large-structure growth that may overwhelm the host body.

Abnormal Colonization by *Cyberlindnera jadinii*: Among the many failed experiments to create artificial symbioses, there was one exception—*Cyberlindnera jadinii* (e.g., nutritional yeast, Torula, syn. *Torulopsis utilis*). Replacement of *Symbiotaphrina* with *Cyberlindnera*, caused widespread infection in both mycetocytes and pillar cells, as well as the remaining midgut epithelial cells (Figure 4b,c). Moreover, this infection did not trigger loss of the brush border [103]. Larvae with this widespread, pathogen-like infection were still capable of developing into adults. Remarkably, if beetles were provided *Symbiotaphrina* and *Cyberlindnera* simultaneously, the widespread infection did not occur, suggesting that the presence of the natural symbiont may immunize the midgut epithelium against infection from foreign fungi [103]. This surprising result even made Paul Buchner question the validity of the taxonomic reclassification of *Symbiotaphrina* (previously *Torulopsis buchnerii*) (Buchner 1965 pg 129) “only *Torulopsis utilis* [*Cyberlindnera jadinii*] was taken up by the sterile mycetocytes and was able to replace the normal symbionts, appear to speak against this new classification [as *Symbiotaphrina*]” [38]. Astoundingly, *Symbiotaphrina* and *Cyberlindnera* share a most recent common ancestor >500 Mya, yet they are both capable of intracellularly infecting *St. paniceum* midgut cells [53]. The recent discovery and isolation of diverse, free-living *Symbiotaphrina* species from plants provides an exciting opportunity to create host–fungus pairs with closely related, but non-symbiotic species (Table 3) [95].

## 4. The Emerging Model System: Advantages and Future Possibilities

### 4.1. Laboratory Rearing and Experimental Manipulation

The same life history aspects that make Drugstore and Cigarette beetles difficult to control pests make them well suited for laboratory rearing and experimental manipulation. These beetles can survive in a wide range of abiotic and dietary conditions, which has allowed them to reach a cosmopolitan distribution. Developmental time, adult survival, and female reproductive output are greatly affected by rearing circumstances; paramount among these conditions are temperature and humidity. Midcentury empirical surveys reared beetles across a range of temperatures and humidities to produce amazingly detailed isopleth maps, identifying that both species reach minimum time to eclosion, while maintaining high survival rates and female egg number near 30 °C and 60–70% rh [58,60,63,64,148].

Evident from the variety of products attacked, these beetles are capable of developing on a wide array of dried plant and animal products (e.g., flour, red pepper, spices, leather, books, textiles, wood) [58]. To promote maximum growth, laboratory colonies are generally maintained on a combination of flour, cornmeal and yeast extract product (i.e., brewer’s or baker’s yeast). Nutritional- or Torula yeast, *Cyberlindnera jadinii*, is not recommended for rearing because of the consequences referenced in Foeckler (1961) and discussed above. A population of 100–300 adults are placed in a screw-top mason jar with 100 g of diet mix and 2 sheets of paper towel (cut into ~7 cm rounds) for adults to congregate [149,150]. The diet should be lightly compressed before the adults are added to produce a harder diet surface for adults to walk on, because they can easily become stuck in loose flour.

After a colony is established, large populations of both species can be kept with minimal hands-on work by having multiple jars of beetles asynchronously developing and maintained by moving a cohort of adults to a new container weekly. In this way, all developmental stages can be acquired within ~3 days. Adults can be collected directly from jars, larvae and pupae can be sifted from the diet, and eggs can be collected from adults. Extensive testing by earlier researchers found that egg collection is best achieved using black paper, folded to create a crevice that females prefer to oviposit into over many different types of materials [58,148]; however, eggs may be sifted from flour as well. Females lay the greatest number of eggs within their first 4 days of life outside the cocoon [58,148]. Over their full life, *St. paniceum* and *L. serricorne* females lay approximately 50 and 100 eggs, respectively, when reared in standard lab conditions [58,60,63,64].

Larvae of both species have high survival rates when reared individually [151,152]. Single eggs placed in 96-well plates with 100 mg of diet produced adults 92% of the time [152]. This rearing method can eliminate larval competition that might interfere with results and also allows for large numbers of replicates and highly adaptable platform to expose larvae to different conditions during experiments. Because larvae prefer to spin cocoons attached to surfaces, many pupae can be sexed looking at the bottom of the plate.

Although it has not been performed before, it may be possible to rear these beetle species without their partner indefinitely. This could be achieved in completely axenic conditions using autoclaved containers fitted with lids to prevent contamination and sterilized diet (gamma-irradiated). Additionally, beetles could be maintained specific-symbiont-free (SSF), similar to specific-pathogen-free (SPF) rearing methods currently employed for mice and other model organisms. These SSF beetles would lack *Symbiotaphrina* but would encounter microorganisms present in diet. However, the long-term effects on the host still needs to be assessed.

Defined diets are available for these beetles [65,82,153,154]. A complete, chemically-defined (holidic) diet is attainable because of the research performed by previous scientists. Such a diet will afford better control in variation for future experiments to see how host and symbiont contribute to digestion and toxin degradation, similar to the system available in *Drosophila*’s holidic diet [155]). These beetles are able to survive on diets composed of protein (casein), carbohydrate (glucose), sterol (cholesterol), salt mix (McCollum’s no. 185), and B-complex vitamins. With this composite diet, individual components can be omitted to measure their effects on host–symbiont interactions.

### 4.2. Modern Methodological Advances

Modern methods provide incredible opportunities for these systems, both in describing the mechanisms underlying host–symbiont interaction at a genetic level and for functionally testing hypotheses by manipulating gene expression.

Genomic Resources: Assembling a quality genome with detailed annotations is a necessary foundation for any research organism or model system. They provide critical sequence data required for numerous techniques to explore diverse research paths. Transcriptomics (normal, single cell, and spatial) allow studies into varied aspects of host–symbiont interactions at the fine-scale, molecular level. Whereas transcriptome sequencing of host-associated bacteria is often difficult in small-bodied invertebrates, which requires separate preparation and rRNA depletion, transcriptomics is easier where both host and symbiont are eukaryotes, since the expression of both partners can be assessed in a single run [156]. Further, a plethora of other ‘omics techniques (e.g., metabolomics, proteomics, lipidomics, glycomics) will add to the detailed biology of and metabolite exchange between host and symbiont.

Functional analyses: The ability to perform functional analyses is critical for a model system exploring the genetic basis of traits. Gene knockdown with RNAi is a widely used technique allowing the quick and inexpensive interrogation of individual gene function. Efficient and systemic knockdown has been observed in all species of beetles where RNAi has been attempted [157]. Recently, highly effective RNAi knockdown was achieved in both *L. serricorne* and *St. paniceum* [151,158], providing proof-of-concept that genetic manipulation is possible in these emerging model organisms.

The powerful tool, CRISPR is becoming available to new and diverse organisms. The anobiid–fungus system is well suited for CRISPR because of the low maintenance required to maintain lines and high-throughput rearing methods. Similar to *Drosophila*, mutant or transformed lines can be maintained by a single research technician individually in a small amount of space using only minimally expensive media.

Phylogenetics analyses: Because of their relatively small genome sizes and the rapidly decreasing cost and effort required for DNA sequencing, whole-genome analysis and phylogenies based on genome-wide orthologs are revolutionizing our understanding of the fungal tree of life [159]. Not only can whole genomes provide insight to relationships between species, they can also reveal the history of individual genes (e.g., duplication, loss). Lateral gene transfers are now commonly identified among fungal genomes, especially in vitamin synthesis pathways and other functional traits possibly associated with ecological transitions (e.g., host-associated symbiosis) [160].

The relationships among Ptinidae *s.l.* beetles are not well understood and they have no detailed phylogenetic tree. Utilizing multigene approaches (e.g., multilocus sequence typing, MLST) may be possible; however, new sequencing technologies may be better suited (e.g., double digest restriction-site associated DNA sequencing, ddRADseq; ultraconserved element phylogenomics, UCEs). UCEs methods were recently used to resolve the second largest suborder of beetles, Adephaga, which shows the power and feasibility of this method [161,162].

While the Drugstore and Cigarette beetle—*Symbiotaphrina* systems offer excellent laboratory models, there are a number of future possibilities exploring the relationship between symbiont diversity and host diversity among the Ptinidae *s.l.* and the broader diversity of Bostrichoidea.

## 5. Beyond the Symbioses of Drugstore and Cigarette Beetles

While the vast majority of our knowledge about the microbial associates of beetles within the superfamily Bostrichoidea comes from *St. paniceum* and *L. serricorne*, there are actually many other bostrichoids that depend upon microbial symbionts. However, few species have been surveyed for symbionts relative to the overall diversity found in this group of beetles. Research directed across bostrichoid diversity will add an evolutionary perspective to the Drugstore and Cigarette beetle systems and, potentially fungal symbioses generally.

### 5.1. Bostrichoid Beetle Taxonomy and Diversity

The superfamily Bostrichoidea is a relatively old group of beetles (~250 My) that includes skin-, powderpost-, spider-, and deathwatch beetles (Figure 3a), which are divided into four families: Endecatomidae, Dermestidae, Bostrichidae, Ptinidae *s.l.* (Ptinidae *s.s. +* Anobiidae *s.s.*). These beetles are generally considered to be small bodied (2–5 mm), morphologically homogeneous, and ecologically static (Figure 3a) [49,163,164,165]. Counter to this narrative, many species specialize on extremely different plant and animal materials (grains, stored products, wood, woody fungi, dung, dried animal carcasses) (Figure 3b), which can have metabolites or toxins that make them difficult to digest [49,166,167]. Additionally, there has been multiple independent transitions to myrmecophily (ant-association) within Ptinidae *s.s.* and possibly Anobiidae *s.s.* (*Fabrasia*) [48,168,169]. Further, while the majority of species are small, the largest member of Bostrichoidae, the giant palm borer—*Dinapate wright*—reaches 5 cm in length and lives in remote palm oases of southern California deserts in *Washingtonia* palms [170].

Bostrichoidea contains a wide variety of species with diverse ecologies that make them important pests in diverse products (Figure 3a,b). Ancestrally associated with live or dead wood [46,48,165], many bostrichoid species have evolved to become pests of products important to humans. Several genera destroy grains (e.g., *Rhyzopertha*, *Prostephanus*) and additional dried products (e.g., *Lasioderma*, *Stegobium*), while other genera attack wood found in furniture and other hardwood materials (flooring, paneling, molding, doorframes) (e.g., *Lyctus*, *Anobium*, *Xestobium*), and softwood housing materials (pallets, pine studs) (e.g., *Sinoxylon*, *Hemicoelus*). Dermestidae beetles (skin beetles) feed on dried animal and plant products and can cause major damage to textiles (carpet, rugs, leatherworks, woven art) (*Dermestes*) [163,171,172,173]. *Dermestes maculatus* alone, was responsible for destroying an estimated 20% of India’s silk production in 1987 [174]. Even literature cannot escape bostrichoids—a few species are able to thrive on the internal pages of books and are thought to be the original “bookworms” [175]. *Tricorynus herbarius* (Mexican book beetle) in particular is especially damaging, in one library it was responsible for damaging nearly 66% of the rare book collection [176]. Altogether, Bostrichoidea represents one of the most destructive lineages of insects, not only to food supplies, but also to human cultural artifacts and can become incredibly destructive pests of museum and herbarium collections, destroying historical documents, wood products and textiles [35,57]. Certain members are known to bore through soft metals such as aluminum, silver, and lead to get at food sources [177]. Of these metal borers, *Scobicia declivis*, has become known as the Lead-Cable borer or Short-Circuit beetle because of its common habit of boring into cables and the resulting power and telephone outages in California and other regions across its native range of western North America. Incredibly, these beetles are also known as the Barrel-Boring beetle because they are also known to attack wine-filled casks, especially in Sonoma county, California [177]. However, in spite of the many harmful aspects of Bostrichoidea, they also contribute invaluable services to nutrient cycling in many ecosystems by decomposing of plant and animal materials. Additionally, because of its specialized ability to feed on decomposing remains, *Dermestes maculatus* is used as an indicator for the time of death in forensic investigations and by taxidermists and natural history museums to clean bone specimens [178].

With their astonishing innate biological capabilities, certain bostrichoids have become the subject of human imagination. Species can take over 10 years to develop from egg to adult, rivaling the life span of 13 year cicadas [179]. These long-lived beetles that inhabit the hardwood features of houses were the inspiration for the Deathwatch legend. Larval *Xestobium rufovillosum* (the Deathwatch beetle) silently develop in wood beams taking ~10 years to reach adulthood and over long time periods can cause major structural damage to historic buildings (churches, etc.). Adult Deathwatch beetles make ticking sounds by knocking their head repeatedly onto wood in a call and response between males and females during courtship [180,181]. Centuries ago, people heard these ticks while silently tending to ill or otherwise near-death relatives in their houses. It is unclear exactly where the legend started, but these ticking beetles were superstitiously assigned to foretell impending death [182]. It is not hard to imagine their rapping as Death’s macabre timer counting down one’s life, or as the expectant tapping of the Grim Reaper’s skeletal fingers as he waits to collect his next soul. The Deathwatch myth, in particular, has been inspiration for many writers as a ghoulish trope used by Mark Twain [183], Henry David Thoreau [184], Ray Bradbury [185], and John Keats [186]. Most notably, the Deathwatch myth was likely an inspiration for Edgar Allen Poe’s short story “The Tell-Tale Heart”, with the murderer confusing a beetle’s ticking with the beating of his dead victim’s heart [187].

### 5.2. Bostrichoid Symbioses

There is wide consensus that Bostrichidae, Ptinidae *s.s.* and Anobiidae *s.s.* form a monophyletic group estimated at ~175 My (Figure 3a) and, while no members of Ptinidae *s.s.* have been surveyed, both Bostrichidae and Anobiidae *s.s.* species commonly harbor microbial symbionts [46,48,49]. Bostrichidae harbor a *Sulcia*-like bacterial symbiont that confers desiccation resistance by altering cuticle thickness, melanization, and cuticular hydrocarbons [188]. Among Anobiidae *s.s.* beetles, there are at least three different fungal symbionts: *Symbiotaphrina* (*St. paniceum*, *L. serricorne*), *Nakazawaea* (*Ernobius mollis, Ernobius abietis*), and *Meyerozyma* (*Xestobium plumbeum*) (Table 6) [37,42,81,102,112,189,190]. More host–symbiont associations have been observed within Anobiidae *s.s.* by dissection and microscopy in the early 20th century but have not been identified to species or followed up with DNA sequencing methods (Table 6).

The three fungal symbionts identified with DNA-based methods in ptinids were discovered from only five beetle species, suggesting that this group of insects may have a high rate of symbiont replacement. Symbiont turnover might be associated with the intracellular/extracellular nature of these symbioses; however, more surveys are needed to understand host–symbiont associations and symbiont transmission across Ptinidae *s.l.* diversity. While *Symbiotaphrina* is a member of the Pezizomycotina, the other symbionts found in Ptinidae *s.l.* (*Nakazawaea*, *Meyerozyma*) are members of the Saccharomycotina, sharing a most recent common ancestor ~520 Mya (Figure 3c) [53]. The extreme taxonomic distance of these symbionts strongly supports the hypothesis that this group of beetles has experienced symbiont replacement, because the divergence age between these symbionts is greater than the age of Bostrichoidea (~250 My) (Figure 3a,c). Surveys for microbial symbionts in Ptinidae *s.l.* and Bostrichidae are required to fully understand the phylogenetics and host–symbiont associations in this group of beetles

Finally, outside of the obligate fungal symbionts found in these beetles, the presence and functional role of other gut-associated microbes (e.g., bacteria) has not been explored. For example, certain cerambicid beetles, which are also ancestrally associated with fungal symbionts, have both a fungal and a bacterial symbiont, while others have only retained the bacterial partner [196,197]. Within Anobiidae *s.s.* symbioses, bacteria may supply or help supply nutrients to the host beetle or fungal symbiont but have been missed because no culture-independent analyses have been performed to examine bacterial communities. Amplicon, metagenomic, and whole-genome sequencing will provide insight into these questions.

## 6. Conclusions

The Drugstore and Cigarette beetles with their symbiont *Symbiotaphrina* provide unique platforms for research on animal–fungus symbiosis across both extracellular and intracellular interactions at a time when fungal diseases and drug resistance are increasing worldwide. The ability to separately maintain the host and the symbiont and produce novel host–symbiont pairs is nearly unparalleled in animal intracellular mutualist systems and provides an opportunity for diverse research possibilities, including to identify factors associated with mutualistic vs. pathogenic intracellular invasion. Building upon the robust historical research, contemporary genomic and functional methodologies promise to quickly advance these systems into modernized model organisms. For example, I was able to make novel hypotheses about the mechanisms underlying differences in thiamine and biotin biosynthesis in *Sy. buchneri* and *Sy. kochii* by combining metabolic information (collected from culture-based tests conducted over half a century ago) with whole-genome sequence data. I foresee many rapid insights coming from this combination of historic perspective and modern technology. Revisiting historic literature is an often-overlooked aspect of academic study, especially with the pressure of staying at the forefront of methodological advances; however, looking back can provide perspective and different insight to current research topics.

As the old metaphor goes, *we stand on the shoulders of giants*—unless we have ignored or lost those giants. The beetle–fungus symbioses presented here have largely fallen into obscurity and the dusty pages of difficult to locate journals; however, these tiny beetles—because of the researchers that advanced our understanding of their biology—are poised to become models of animal–fungus interactions. Reviving these forgotten systems will stimulate research spanning mutualism, pathogenesis, the microbiota, and applied pest management.

## Figures and Tables

**Table 1:** Development time for beetles.

	*Stegobium paniceum*	*Lasioderma serricorne*
Egg	7	7
Larva	36	18
Pupa	4	4
Adult	14	20
Eggs/Female	50	100

Approximate # days for beetles reared at 30 °C, 60–70% r.h. Azab (1943) [60], Kashef (1956) [63], Howe (1957) [64], Lefkovitch (1967) [58], Lefkovitch & Currie (1967) [65].

**Table 2:** Metabolism of *Symbiotaphrina* on culture media.

		*Sy. buchneri*	*Sy. kochii*
Carbon Assimilation	Glucose	+	+
	Galactose	+	+
	Sucrose	−	+
	Maltose	+	+
	Lactose	−	−
	L-Sorbose	+	+
	Cellobiose	+	+
	Melibiose	−	+
	Raffinose	+	+
	Melezitose	+	+
	D-Xylose	+	+
	L-Arabinose	+	+
	D-Arabinose	−	+
	Ribose	+	+ (slow)
	Rhamnose	+ (slow)	+ (slow)
	Ethanol	−	− (+)
	Erythritol	+	+
	Adonitol/Ribitol	−	+
	Dulcitol/Galactitol	+	+
	Mannitol	+	+
	Sorbitol	+	+
	Methyl-D-glucoside	+	+ (slow)
	Salicin	−	+
	Lactic acid	−	+ (slow)
	Succinic acid	+	+ (+ slow)
	Citric acid	+	+ (−)
Nitrogen Assimilation	Ammonium sulfate	+	+
	Potassium nitrate	+	−
	Urea	−	−
	Asparagine	+	+
Other aspects	Pseudomycelium formation	none	none
	Spores	none	none
	Fermentation	none	none
	Arbutin cleavage	none	positive
	Vitamin requirements	thiamine, biotin	none
	Growth at 37 °C	none	none
	Colony morphology (dark)	white, soft, surface smooth, glossy	white, soft, surface smooth, glossy
	Grown in light	red	red

Data from Kühlwein and Jurzitza (1961) [84] (*Sy. buchneri*) and Jurzitza (1964) [86] (*Sy. kochii*).

**Table 3:** Sexual reproduction differences among *Symbiotaphrina* species.

	Asexual Morph			Sexual Morph	Isolation Location		Culture Available
*Symbiotaphrina* sp.	Yeast like	Mycelia	Conidia	Apothecia			
*buchneri*	Yes	No	No	No	Drugstore beetle (*Stegobium paniceum*)	Mycetome, eggs	Yes (Many)
*kochii*	Yes	No	No	No	Cigarette beetle (*Lasioderma serricorne*)	Mycetome, eggs	Yes (Many)
*lignicola*	Yes	Yes	Yes	No	Aspen (*Populus tremuloides*)	Living tree (galls, cankers)	Yes (CBS 325.93)
*sanguinea*	Yes	Yes	Yes	No	Oak and Chestnut (*Quercus* and *Castanea*)	Tanning liquid (for leather)	Yes (CBS 406.52)
*desertorum*	Yes*	Yes	Yes	Yes	*Krascheninnikovia, Purshia, Acacia*	Decayed wood	No
*microtheca*	Yes	Yes	Yes	Yes	Conifers (*Pinus, Picea, Abies*)	Decayed wood	Yes (CBS 110481, 82, 83)
*larreae*	–	–	–	Yes	Creosote (*Larrea tridentata*)	Decayed wood	No

* yeast-like growth without mycelia was observed on MEA media Baral et al. (2018) [95].

**Table 4:** Comparative genomics among Xylonomycetes.

	*Sy. kochii*	*Sy. buchneri*	*X. heveae*	*T. guianense*
Genome Size (Mb)	25.19	24.01	24.69	24.57
# Scaffolds	54	169	27	236
GC (%)	50.85	50.97	47.38	47.09
Predicted CDS #	10482	9367	8205	8062
Avg. CDS size (bp)	1377	1450	1487	1473
Seq. Center	JGI	RIKEN	JGI	JGI
Host	*L. serricorne*	*St. paniceum*	Free living	Free living

**Table 5:** B vitamin biosynthesis pathway (thiamine and biotin) comparison among Xylonomycete genomes.

Pathway	KO #	Gene Name	*Sy. kochii*	*Sy. buchneri*	*X. heveae*	*T. guianense*
Thiamine	K06215	snz1	1	1	1	1
Thiamine	K08681	sno1	1	1	1	1
Thiamine	K00868	Bud16	1	1	1	1
Thiamine	K00275	pdx3	2	3	2	2
Thiamine	K18278	Thi5	1	–	1	1
Thiamine	K00877	Thi20	1	1	1	1
Thiamine	K03146	Thi4/Thi1	1	1	1	1
Thiamine	K00788	Thi6	1	1	1	1
Thiamine	K14154	Thi6 bifunctional	1	1	1	1
Thiamine	K01078	3.1.3.2	5	3	2	2
Thiamine	K00949	Thi80	1	1	1	1
Biotin	K01906	Bio1	–	–	–	–
Biotin	K00652	Bio6	–	–	–	–
Biotin	K00833	Bio3	–	–	–	–
Biotin	K01935	Bio4	–	–	–	–
Biotin	K19565	Bio5	0 (23)*	0 (17)*	0 (9)*	0 (9)*
Biotin	K19562	Bio3-Bio1 bifunctional	1	–	1	1
Biotin	K01012	Bio2	1	–	1	1

* Bio5 is not identified, but each genome contains several high sequence similarity choline transporters (K19564) in parentheses. One or more of these likely functions as a KAPA transporter.

**Table 6:** Beetle–microbe symbioses observed in Bostrichoidea.

Beetle					Symbiont								
Genus	Species	Superfamily	Family	Subfamily	Domain/ Kingdom	Subphylum/ Phylum	Genus	Species	Evidence	Symbiont Description	Culture Attempted	Culture Available	Citation
*Anobium*	*emarinatum*	Bostrichoidea	Anobiidae	Anobiinae	Fungi?	–	–	–	Visual	Teardrop shaped	Fail	–	[81]
*Anobium*	*hederae*	Bostrichoidea	Anobiidae	Anobiinae	Fungi?	–	–	–	Visual	oval to long-oval	Fail	–	[189]
*Anobium*	*pertinax*	Bostrichoidea	Anobiidae	Anobiinae	Fungi?	–	–	–	Visual	Teardrop shaped	Fail	–	[81,190]
*Anobium*	*punctatum*	Bostrichoidea	Anobiidae	Anobiinae	Fungi?	–	–	–	Visual	Oval to cylindrical rarely teardrop	Fail	–	[42,81,112,190]
*Anobium*	*striatum*	Bostrichoidea	Anobiidae	Anobiinae	Fungi?	–	–	–	Visual	Elongated, oval to cylindrical	Fail	–	[42,81,112,190]
*Cacotemnus*	*rufipes*	Bostrichoidea	Anobiidae	Anobiinae	Fungi?	–	–	–	Visual	Very large yeast	–	–	[42,81]
*Hemicoelus*	*fulvicorne*	Bostrichoidea	Anobiidae	Anobiinae	Fungi?	–	–	–	Visual	–	–	–	[42,81]
*Hemicoelus*	*nitidum*	Bostrichoidea	Anobiidae	Anobiinae	Fungi?	–	–	–	Visual	Long, oval, partly teardrop, few Giant cells	Fail	–	[42,81]
*Oligomerus*	*brunneus*	Bostrichoidea	Anobiidae	Anobiinae	Fungi?	–	–	–	Visual	large, oval or pointed on one side	Fail	–	[81,42,189]
*Priobium*	*carpini*	Bostrichoidea	Anobiidae	Anobiinae	Fungi?	–	–	–	Visual	Spherical to oval	Fail	–	[42,189]
*Stegobium*	*paniceum*	Bostrichoidea	Anobiidae	Anobiinae	Fungi	Pezizomycotina	*Symbiotaphrina*	*buchneri*	Culture/Sequence	Teardrop shaped	Succeed	Multiple	[83,84]
*Anitys*	*rubens*	Bostrichoidea	Anobiidae	Dorcatominae	Fungi?	–	–	–	Visual	–	–	–	[81,191]
*Caenocara*	*bovistae*	Bostrichoidea	Anobiidae	Dorcatominae	Fungi?	–	–	–	Visual	–	–	–	[81,191]
*Dorcatoma*	*chrysomelina*	Bostrichoidea	Anobiidae	Dorcatominae	Fungi?	–	–	–	Visual	–	–	–	[81,191]
*Dorcatoma*	*dresdensis*	Bostrichoidea	Anobiidae	Dorcatominae	Fungi?	–	–	–	Visual	Roundish, rarely somewhat pointed, small	–	–	[81,191]
*Dorcatoma*	*flavieornis*	Bostrichoidea	Anobiidae	Dorcatominae	Fungi?	–	–	–	Visual	–	–	–	[81,191]
*Dorcatoma*	*serra*	Bostrichoidea	Anobiidae	Dorcatominae	Fungi?	–	–	–	Visual	–	–	–	[81,191]
*Dorcatoma*	*setosella*	Bostrichoidea	Anobiidae	Dorcatominae	Fungi?	–	–	–	Visual	–	–	–	[81,191]
*Stagetus*	*pellita*	Bostrichoidea	Anobiidae	Dorcatominae	Fungi?	–	–	–	Visual	–	–	–	[81,191]
*Stagetus*	*pilula*	Bostrichoidea	Anobiidae	Dorcatominae	Fungi?	–	–	–	Visual	–	–	–	[81,191]
*Grynobius*	*planus*	Bostrichoidea	Anobiidae	Dryophilinae	Fungi?	–	–	–	Visual		Fail	–	[81,189]
*Ernobius*	*abietis*	Bostrichoidea	Anobiidae	Ernobiinae	Fungi	Saccharomycotina	*Nakazawaea^1^*	*ernobii*	Culture/Sequence	Roundish to oval	Succeed	NRRL, Y-17655	[81,112,189,190]
*Ernobius*	*mollis*	Bostrichoidea	Anobiidae	Ernobiinae	Fungi	Saccharomycotina	*Nakazawaea^2^*	*ernobii*	Culture/Sequence	Round yeast	Succeed	NRRL, Y-12940	[81,189,190]
*Ernobius*	*nigrinus*	Bostrichoidea	Anobiidae	Ernobiinae	Fungi?	–	–	–	Visual	–	Fail	–	[81,189]
*Xestobium*	*plumbeum*	Bostrichoidea	Anobiidae	Ernobiinae	Fungi	Saccharomycotina	*Meyerozyma^3^*	*carpophila*	Culture/Sequence	Roundish & small, spherical bacteria (in other gut cells!)	Succeed	NRRL, Y-17685	[81,189]
*Xestobium*	*rufovillosum*	Bostrichoidea	Anobiidae	Ernobiinae	Fungi?	–	–	–	Visual	Short-oval, Round with one or two lobes (lemon shaped)	Fail	–	[81,112,189,190]
*Hedobia*	sp.	Bostrichoidea	Anobiidae	Eucradinae	–	–	None / Doubtful	–	Visual	–	–	–	[42,81]
*Mesocoelopus*	*collaris*	Bostrichoidea	Anobiidae	Mesocoelopodinae	Fungi?	–	–	–	Visual	–	–	–	[81,189,191]
*Mesocoelopus*	*niger*	Bostrichoidea	Anobiidae	Mesocoelopodinae	Fungi?	–	–	–	Visual	Small, slim, somewhat pointed on one side	Fail	–	[81,191]
*Ptilinus*	*fuscus*	Bostrichoidea	Anobiidae	Ptilininae	–	–	Doubtful	–	Visual	–	Fail	–	[81,192]
*Ptilinus*	*pectinicornis*	Bostrichoidea	Anobiidae	Ptilininae	Fungi?	–	Yeast ?	–	Visual	Large, teardrop shaped	Fail	–	[81,189]
*Lasioderma*	*redtenbacheri*	Bostrichoidea	Anobiidae	Xyletininae	–	–	None	–	Visual	–	Fail	–	[42,81,189]
*Lasioderma*	*serricorne*	Bostrichoidea	Anobiidae	Xyletininae	Fungi	Pezizomycotina	*Symbiotaphrina*	*kochii*	Culture/Sequence	Teardrop shaped	Succeed	Multiple	[83,84]
*Xyletinus*	*ater*	Bostrichoidea	Anobiidae	Xyletininae	–	–	None	–	Visual	–	–	–	[42,81]
*Xyletinus*	*pectinatus*	Bostrichoidea	Anobiidae	Xyletininae	–	–	None	–	Visual	–	–	–	[42,81,189]
*Apate*	*degener*	Bostrichoidea	Bostrichidae	Bostrichinae	Bacteria	Bacteroidetes	*Sulcia*-like	sp.	Visual	–	–	–	[193,194,195]
*Apate*	*monachus*	Bostrichoidea	Bostrichidae	Bostrichinae	Bacteria	Bacteroidetes	*Sulcia*-like	sp.	Visual	–	–	–	[193,194,195]
*Bostrychoplites*	*zickeli*	Bostrichoidea	Bostrichidae	Bostrichinae	Bacteria	Bacteroidetes	*Sulcia*-like	sp.	Visual	–	–	–	[193,194,195]
*Scobicia*	*chevrieri*	Bostrichoidea	Bostrichidae	Bostrichinae	Bacteria	Bacteroidetes	*Sulcia*-like	sp.	Visual	–	–	–	[193,194,195]
*Sinoxylon*	*ceratoniae*	Bostrichoidea	Bostrichidae	Bostrichinae	Bacteria	Bacteroidetes	*Sulcia*-like	sp.	Visual	–	–	–	[193,194,195]
*Sinoxylon*	*sexdentatus*	Bostrichoidea	Bostrichidae	Bostrichinae	Bacteria	Bacteroidetes	*Sulcia*-like	sp.	Visual	–	–	–	[193,194,195]
*Dinoderus*	*bifoveolatus*	Bostrichoidea	Bostrichidae	Dinoderinae	Bacteria	Bacteroidetes	*Sulcia*-like	sp.	Sequence	–	–	–	[188]
*Dinoderus*	*porcellus*	Bostrichoidea	Bostrichidae	Dinoderinae	Bacteria	Bacteroidetes	*Sulcia*-like	sp.	Sequence	–	–	–	[188]
*Dinoderus*	sp.	Bostrichoidea	Bostrichidae	Dinoderinae	Bacteria	Bacteroidetes	*Sulcia*-like	sp.	Sequence	–	–	–	[188]
*Prostephanus*	*trunctatus*	Bostrichoidea	Bostrichidae	Dinoderinae	Bacteria	Bacteroidetes	*Sulcia*-like	sp.	Sequence	–	–	–	[188]
*Rhyzopertha*	*dominica*	Bostrichoidea	Bostrichidae	Dinoderinae	Bacteria	Bacteroidetes	*Sulcia*-like	sp.	Sequence	–	–	–	[195,188]
*Lyctus*	*brunneus*	Bostrichoidea	Bostrichidae	Lyctinae	Bacteria	Bacteroidetes	*Sulcia*-like	sp.	Sequence	–	–	–	[188]
													

Original names: ^1^ *Candida karawaiewii;* ^2^ *Candida ernobii;* ^3^ *Candida xestobii* Breitsprecher (1928) [42]; Buchner (1954) [194]; Engl et al. (2018) [188]; Gräbner (1954) [81]; Heitz (1927) [112]; Jurzitza (1970) [189]; Kühlwein & Jurzitza (1961) [84]; Mansour (1934) [193]; Müller (1934) [190]; Nolte (1938) [191]; Okude et al. (2017) [195]; Pant & Fraenkel (1954) [83]; Schanderl (1942) [192].
